# A cohort study to identify risk factors for *Plasmodium falciparum* infection in Burkinabe children: implications for other high burden high impact countries

**DOI:** 10.1186/s12936-020-03443-x

**Published:** 2020-10-16

**Authors:** Jean Baptiste Yaro, Alphonse Ouedraogo, Z. Amidou Ouedraogo, Amidou Diarra, Malik Lankouande, Efundem Agboraw, Eve Worrall, Kobié Hyacinthe Toe, Antoine Sanou, W. Moussa Guelbeogo, N’Fale Sagnon, Hilary Ranson, Alfred B. Tiono, Steven W. Lindsay, Anne L. Wilson

**Affiliations:** 1 Centre National de Recherche et de Formation sur le Paludisme, Ouagadougou, Burkina Faso; 2 Department of Biosciences, Durham University, Durham, UK; 3 Department of Vector Biology, Liverpool School of Tropical Medicine, Liverpool, UK; 4 Institute of Biodiversity Animal Health & Comparative Medicine, Glasgow University, Glasgow, UK

**Keywords:** Malaria, Epidemiology, Cohort study, Burkina faso, Vector control, Insecticide resistance

## Abstract

**Background:**

Progress in controlling malaria has stalled in recent years. Today the malaria burden is increasingly concentrated in a few countries, including Burkina Faso, where malaria is not declining. A cohort study was conducted to identify risk factors for malaria infection in children in southwest Burkina Faso, an area with high insecticide-treated net (ITN) coverage and insecticide-resistant vectors.

**Methods:**

Incidence of *Plasmodium falciparum* infection was measured in 252 children aged 5 to 15 years, using active and passive detection, during the 2017 transmission season, following clearance of infection. Demographic, socio-economic, environmental, and entomological risk factors, including use of ITNs and insecticide resistance were monitored.

**Results:**

During the six-month follow-up period, the overall incidence of *P. falciparum* infection was 2.78 episodes per child (95% CI = 2.66–2.91) by microscopy, and 3.11 (95% CI = 2.95–3.28) by polymerase chain reaction (PCR). The entomological inoculation rate (EIR) was 80.4 infective bites per child over the six-month malaria transmission season. At baseline, 80.6% of children were reported as sleeping under an ITN the previous night, although at the last survey, 23.3% of nets were in poor condition and considered no longer protective. No association was found between the rate of *P. falciparum* infection and either EIR (incidence rate ratio (IRR): 1.00, 95% CI: 1.00–1.00, *p* = 0.08) or mortality in WHO tube tests when vectors were exposed to 0.05% deltamethrin (IRR: 1.05, 95% CI: 0.73–1.50, *p* = 0.79). Travel history (IRR: 1.52, 95% CI: 1.45–1.59, *p* < 0.001) and higher socio-economic status were associated with an increased risk of *P. falciparum* infection (IRR: 1.05, 95% CI: 1.00–1.11, *p* = 0.04).

**Conclusions:**

Incidence of *P. falciparum* infection remains overwhelmingly high in the study area. The study findings suggest that because of the exceptionally high levels of malaria transmission in the study area, malaria elimination cannot be achieved solely by mass deployment of ITNs and additional control measures are needed.

## Background

Malaria remains an acute public health problem throughout sub-Saharan Africa (SSA) with an estimated 213 million cases and 380,000 deaths in 2018 [1]. Despite unprecedented declines in malaria between 2000 and 2015, of which 68% can be attributed to the scale-up of insecticide-treated nets (ITNs) [2], recent years have seen stagnating progress in high burden countries [1]. Reasons for this lack of progress are unclear, but may include incomplete coverage of ITNs, nets in poor condition and malaria vectors resistant to the pyrethroid insecticides used for ITNs. Burkina Faso, along with 10 other high-burden countries in Africa, plus India, has been designated as a High Burden to High Impact country by the World Health Organization (WHO) and the Roll Back Malaria Partnership which calls for an aggressive new approach to accelerate malaria control [3]. Burkina Faso has the seventh highest number of malaria cases globally [1], and has seen a rise in the number of malaria cases from 9 million in 2016 to over 11 million in 2017 and 2018 [4–6].

Despite the known protective efficacy of ITNs, the 2010 ITN universal coverage campaign in Burkina Faso did not decrease malaria and all-cause mortality in children younger than 5 years old, even with 92% of children reportedly sleeping under ITNs [7]. The study site in Burkina Faso has extremely high prevalence and intensity of pyrethroid resistance in malaria vectors [8, 9]. For example, mosquitoes reared from larvae collected in Tengrela village between 2016 and 2018, showed only 2% mortality when exposed to the standard diagnostic dose of deltamethrin of 0.05% designed to kill all mosquitoes [10]. The public health impact of insecticide resistance, however, remains contested [11–14]. Insecticide resistance is only one important factor affecting the efficacy of nets, since ITN coverage, durability and whether people actually sleep under nets, as well as mosquito biting time and behaviour are also important. There is increasing evidence that so-called long-lasting insecticidal nets are not effective for 3 years [15–17], as claimed, and is the reason the term ITN is used. Coverage and system inefficiencies mean that ITNs are unevenly distributed among households and an estimated 50% of ITNs are lost from households after 23 months in Africa [18]. Social and environmental factors, including access to healthcare, socio-economic status (SES) and house construction, found to be protective against malaria in other studies [19], may also impact malaria risk in Burkina Faso.

A cohort study of children was conducted to determine risk factors for *Plasmodium falciparum* infection in an area of persistent and intense malaria transmission in rural Burkina Faso, with high ITN coverage and high levels of insecticide resistance. The study findings may help to identify potential opportunities for improving malaria control in Burkina Faso and other countries in SSA experiencing persistently high malaria transmission.

## Methods

### Study site

**Fig. 1:**
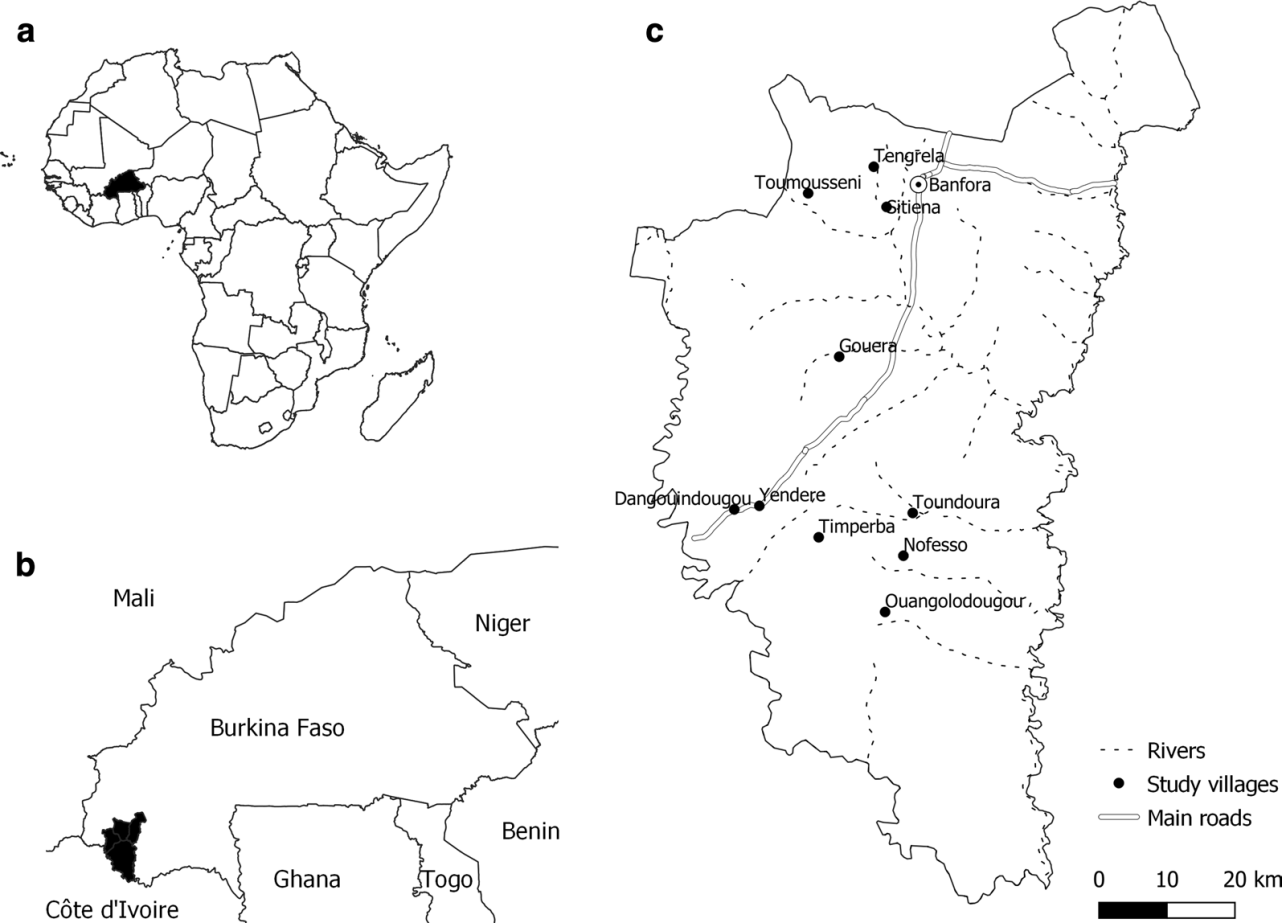
Study location. **a** Location of Burkina Faso; **b** Location of study site in Burkina Faso; **c** Location of the 10 study villages and Banfora (provincial capital) in study site

The study was conducted from June to December 2017 in Banfora Health District, Cascades region, southwest Burkina Faso (Fig. 1), an area of Sudanian savannah covering 6,295 sq km and with an estimated population of 407,073 inhabitants [4]. Subsistence farming and animal husbandry are the main activities. Banfora District has intense seasonal malaria transmission over a six-month period following seasonal rains from May to November [20]. *Plasmodium falciparum* accounts for 90% of cases [20]. The main malaria vector is *Anopheles gambiae *sensu stricto (*s.s*.), but *Anopheles coluzzii* is also found [21]. A universal coverage (defined as one ITN for every two persons at risk of malaria) campaign in 2016 distributed ITNs with permethrin or deltamethrin (Sumitomo Chemical, Vestergaard and BASF) and therefore no new ITNs were distributed by the study. No indoor residual spraying (IRS) was conducted. Since 2014, children under 5 years of age in the study area receive seasonal malaria chemoprevention (SMC) during the transmission season [22].

### Recruitment of study cohort

Ten villages were randomly selected from a list of villages in the study area following a two-stage process. Firstly, an area spanning the catchment areas of five health centres was chosen. Each health centre had a catchment radius of approximately 10 km. Secondly, two villages at least 3 km apart were chosen at convenience from each catchment area, giving a total of 10 villages. Thirty children were randomly selected from each village using the Health and Demographic Surveillance System enumeration list. Children were eligible to participate if they were aged 5–15 years, likely to remain resident in the village for the duration of the study and the caregiver provided informed consent (assent of child if aged 12–15 years). Older children were selected as they have the highest malaria incidence, relatively low immunity and contribute substantially to transmission [23], while children under 5 years were excluded due to roll-out of SMC. Children were not eligible to participate if they were taking part in a malaria clinical trial, or had a contra-indication to the artemisinin-combination therapy (ACT), artemether-lumefantrine (AL). At enrolment in June 2017, 300 children provided a blood film. Irrespective of their malaria parasite status, all children received a curative dose of AL (Wellona Pharma Private Limited, Nana Varachha, Surat, India) to clear any existing parasitaemia. Children were revisited 21 days later, at which time two blood slides and a blood spot were taken and examined to ensure parasite clearance. Those with a negative parasite status confirmed by polymerase chain reaction (PCR) were enrolled in the study. Children re-infected in the 21 day period were re-treated with AL and were eligible for enrolment in the study after 28 days once parasite clearance was confirmed using PCR.

### Follow-up of study cohort

Symptomatic and asymptomatic *Plasmodium* infections were recorded using both active and passive detection. Study children were visited at home every 2 weeks by fieldworkers during the peak transmission season, from July-December 2017. At each visit, fieldworkers measured a child’s axillary temperature and prepared two blood films and a filter paper blood spot. Children with an axillary temperature ≥ 37.5 °C or history of fever in the previous 48 h were advised to visit the local health centre and, if they were a malaria case, treated with AL, following National Malaria Control Programme (NMCP) guidelines [24]. If a child was absent at the time of the visit, the fieldworker made one more attempt to locate them the following day, after which the child was recorded as being absent. Caregivers were encouraged by the study team at enrolment and at fortnightly visits to take their child to the nearest government health centre should the child have a fever or feel unwell. Travel and treatment costs for sick children were reimbursed by the study. Study nurses were posted in the five health centres close to the study villages and performed rapid diagnostic tests for malaria (SD BIOLINE Malaria Ag P.f/Pan, Abbott Laboratories, Illinois, USA) in study children presenting with an axillary temperature ≥ 37.5 °C or history of fever within the previous 48 h, and prepared two blood films and a filter paper blood spot. Children diagnosed with clinical malaria received AL [24]. Clinical data were recorded on dedicated study logs and transcribed by fieldworkers who visited each nurse weekly.

Thick blood films were stained with Giemsa and examined under 1000-fold magnification by experienced microscopists at the *Centre National de Recherche et de Formation sur le Paludisme* (CNRFP) in Banfora. Parasite counts were recorded per high power field and 100 fields counted before a slide was declared negative. Two blood slides from each subject were read separately by two microscopists. Discrepancies in positive and negative reads and parasite counts differing by more than tenfold between the two reads were resolved by the supervisor. Filter paper blood samples were analysed for the presence of 18 s rRNA gene using PCR [25].

### Risk factors

At enrolment, sociodemographic characteristics of the child (age, gender, religion, ethnicity) and their caregiver (education, occupation) were recorded. Questionnaires recorded the presence or absence of large domestic animals (cattle, goats, sheep, donkeys, horses) within 5 m of each study child’s house and the materials used to construct the building in which the study child slept, including presence of a metal roof, eaves and door and window screening. The head of household completed a questionnaire on their asset ownership, house construction and other variables, following standard procedures used in the Burkina Faso Malaria Indicator Survey (MIS) [26]. Houses of study children were geolocated using a global positioning system (GARMIN eTrex 20).

Use of an ITN the previous night by each study child and use of spatial or topical repellents were recorded at enrolment and each active visit. Survivorship and integrity of each study child’s ITN was measured in July, October and December. Each net was recorded as being in use (i.e., hanging over the study child’s sleeping space), in storage, being washed, or lost. Loss was categorized as: (i) net given away voluntarily; (ii) net stolen; or, (iii) net destroyed, discarded or used for alternative purposes. Fabric integrity of the net was assessed by counting the number of holes and their size according to WHO guidance [27]. A weighted sum of hole counts, the proportionate hole index (pHI), was calculated with a pHI of 0–64 categorized as good, 65–642 as acceptable and 643 + as too torn and non-protective [28]. To measure ITN bio-efficacy, 26 bed nets were sampled (at least 2 randomly selected nets per village, except for Sitiena village where 1 net was tested) in October 2017 and stored at + 4 °C. ITNs taken for testing were replaced with new ones. WHO cone bioassays were performed using the pyrethroid-susceptible Kisumu strain of *An. gambiae *sensu lato (*s.l.*) at the CNRFP insectaries in Banfora using the WHO efficacy requirement of ≥ 80% mortality [29].

Mosquitoes were sampled with CDC light traps, positioned with the light 1 m above the ground at the foot end of the bed of each study child sleeping under an ITN from 19.00 to 06.00, every 4 weeks from July to December 2017. In addition, each child’s net was systematically searched for mosquitoes between 06.00 and 07.00 every 4 weeks using a torch. Mosquitoes were identified morphologically using established keys and female *An. gambiae s.l.* typed to species using PCR. The presence of sporozoites in *An. gambiae s.l.* was determined using an enzyme-linked immunosorbent assay [30]. Larval surveys to determine the proximity of anopheline larval habitats to study children’s houses were carried out in the vicinity of all 10 villages during September 2017. All types of larval habitats were surveyed including irrigated fields, puddles, muddy foot or hoof prints, streams and ponds.

Phenotypic insecticide resistance was measured using WHO tube tests as per standard procedures [31]. Assays were performed with *An. gambiae s.l.* mosquitoes reared from immatures collected in seven study villages (larvae were not found in the other three villages).

### Data management and statistical analysis

Data were collected on android personal digital assistants programmed using KoboCollect and included drop-down boxes and consistency checks to reduce data entry errors. Following cleaning, the dataset was locked and saved in Microsoft Access. An analytical plan was developed prior to data analysis.

The primary outcome was the incidence rate of microscopically confirmed *P. falciparum* infection during the transmission season, detected using active and passive case detection. PCR-confirmed *P. falciparum* incidence rate was a secondary outcome. After ACT treatment, further infections were censored for 28 days since infections during this time were most likely due to recrudescent parasites. Time at risk was also censored for time that study children spent away from their compound should they be found to be absent at the two-weekly home visits. The entomological inoculation rate (EIR) or estimated number of infectious bites per study child during the transmission season was calculated using the formula EIR = *MaSd* where *Ma* is the human biting rate, estimated from the arithmetic mean number of female *An. gambiae s.l.* caught per light trap night across the six-month transmission season, where *S* is the proportion of female *An. gambiae s.l.* found to be sporozoite positive by village and *d* is the number of days in the transmission season (n). Cone bioassay results for the netting pieces from each sampled net were pooled by village and by net type. QGIS Geographic Information System (QGIS Development Team (2019), Open Source Geospatial Foundation Project) was used to determine distances between the child’s home and aquatic habitats. Distance to the nearest health facility was determined based on the shortest distance to travel by road. Principal component analysis was used to calculate a SES factor score based on asset ownership and household characteristics. SES factor scores (ranging from -1.8 to 3.2) were ranked and households divided into five equal wealth quintiles (1 poorest, through to 5, least poor).

Mean values were compared using a t-test and proportions compared using Chi-squared tests. Poisson regression models were used to identify risk factors associated with *P. falciparum* infection incidence rate, adjusting for clustering by village. Risk factors assessed were: child’s age, gender, ethnic group, religion, caregiver’s education and occupation, wealth quintile and SES factor score, use of ITNs and other personal protection, ITN integrity, number of people sleeping in the room with the study child, housing materials (roof, eaves, wall and floor material, door and window screening), presence of cows, horses, sheep or goats within 5 m of the child’s home, Euclidean distance to the nearest aquatic habitat with larval anophelines, distance by road to the nearest health centre, EIR at village level, and percentage mortality of *An. gambiae s.l.* mosquitoes from the village when exposed to 0.05% deltamethrin in a WHO tube test. A multivariate regression model was developed using a forward stepwise approach. Statistical analysis was carried out in Stata 15 (Statacorp, Texas, USA).

Assuming two *P. falciparum* infections per child during the transmission season, an intraclass correlation coefficient of 0.1 (design effect of 3.4), and a loss to follow-up of 10%, the study had greater than 90% power to detect effect sizes of > 50% at the 5% level of significance, assuming 50% prevalence of the risk factor of interest in the population using the formula for comparison of two rates [32]. The study is reported following STROBE guidelines [33].

## Results

**Fig. 2:**
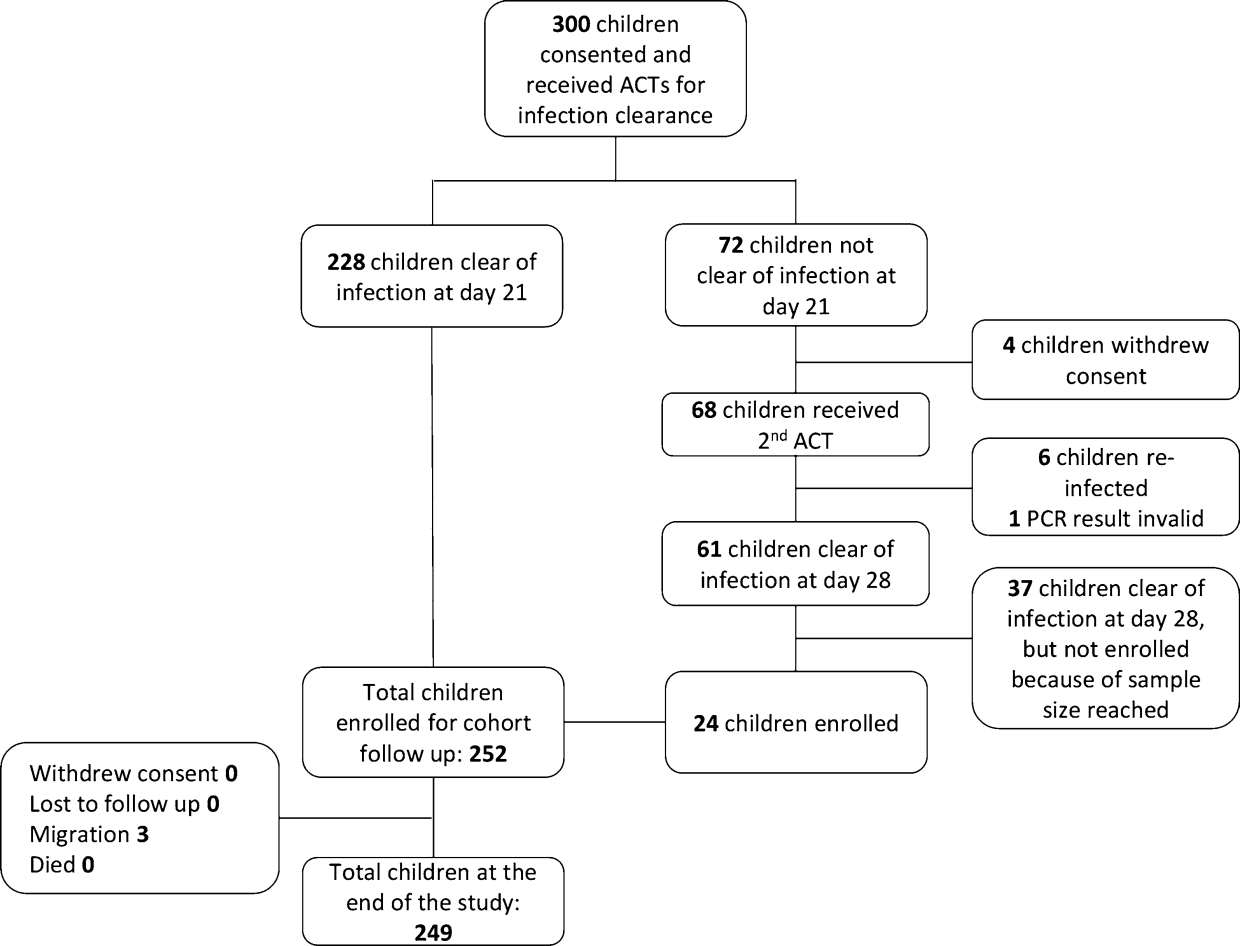
Study flowchart

Of 300 randomly selected children, 252 children aged 5–15 years old were enrolled in the cohort after confirmation of parasite clearance by PCR (Fig. 2). Of these 252 children, 228 were clear of infection at day 21 post-ACT, while an additional 24 children were enrolled following receipt of a second course of ACT due to re-infection. Three children withdrew from the study due to migration. No deaths were recorded among the cohort during the study period.

The mean age of cohort participants was 9.9 years, 52.0% of whom were male (Table 1). 38.9% of children were Gouin, 21.8% Karaboro, 11.5% Mossi, 9.1% Turka, 6.3% Fulani, and 4.4% Senoufo. Caregivers were predominantly illiterate (79.0%) and farmers (95.2%). Most sleeping rooms of the children had metal roofs (75.8%), brick walls (57.9%) and cement floors (70.2%). Over half the children’s sleeping rooms had open eaves (54.8%) and the vast majority did not have window screening (96.0%).

**Table 1:** Baseline characteristics of the study cohort

Characteristic	Number (%) N = 252
Age at enrolment	
5 years to < 8 years	76 (30.2%)
≥ 8 years	176 (69.8%)
Age (mean/standard deviation)	9.93 (2.8)
Gender	
Male	131 (52.0%)
Female	121 (48.0%)
Ethnicity	
Gouin	98 (38.9%)
Karaboro	55 (21.8%)
Mossi	29 (11.5%)
Turka	23 (9.1%)
Fulani	16 (6.3%)
Senoufo	11 (4.4%)
Others	20 (7.9%)
Reported bed net use	
Used bed net usually	215 (85.3%)
Used a bed net the previous night	203 (80.6%)
Caregiver’s education level	
Illiterate	199 (79.0%)
Primary school	45 (17.9%)
Secondary school or above	8 (3.2%)
Caregiver’s occupation	
Not working/retired	6 (2.4%)
Farmer	240 (95.2%)
Commercial activities / government officer	6 (2.4%)
Eave status of child’s sleeping room	
Closed	102 (40.5%)
Open	138 (54.8%)
Roof material of child’s sleeping room	
Metal	191 (75.8%)
Thatch	34 (13.5%)
Other roof type	18 (7.1%)
Wall material of child’s sleeping room	
Mud	65 (25.8%)
Brick	146 (57.9%)
Cement (plastered or painted)	32 (12.7%)
Floor material of child’s sleeping room	
Cement	177 (70.2%)
Dirt floor	65 (25.8%)
Tiles	1 (0.4%)
Window screening of child’s sleeping room	
Absent	242 (96.0%)
Present	1 (0.4%)

During the follow-up period, 249 of the 252 children experienced at least one *P. falciparum* infection, as detected by microscopy. 31 children (12.3%) had one *P. falciparum* infection, 139 (55.2%) children experienced two *P. falciparum* infections, 75 (29.8%) experienced three *P. falciparum* infections, and 4 (1.6%) experienced four *P. falciparum* infections. A total of 550 *P. falciparum* infections were identified using microscopy while 608 infections were identified using PCR. Of the 550 *P. falciparum* infections confirmed using microscopy, 528 (96.0%) were detected using active case detection and 22 (4.0%) detected using passive case detection. Infections detected passively had a higher geometric mean *P. falciparum* density (19,875 mL, 95% CI = 7896–31,854) compared to those detected through active surveillance (3744 mL, 95% CI = 2,691–4,797, p < 0.001). The *P. falciparum* infection incidence rate was 2.78 episodes per child during the six-month transmission season (95% CI = 2.66–2.91) by microscopy, and 3.11 episodes (95% CI = 2.95–3.28) by PCR. Among children suffering from at least one infection, the median time to first infection detected by microscopy was 27 days (range 14–123 days).

At baseline, 80.6% of caregivers reported that the study child slept under an ITN the previous night. Reported ITN use the previous night at the baseline survey was greater than 82% across the four lowest SES quintiles compared to the least poor children (quintile 5 = 53.2%, *p* < 0.001). Most study children’s bed nets were either Permanet 2.0 (52.0%) or Olyset net (16.7%), with 85.5% provided by the NMCP and 4.0% purchased on the open market. Sleeping place inspections in July, October and December found 87.8% of children had a bed net hanging over their sleeping place (638 out of 727 observations). The most common reason for not having a net hanging was loss of the net (59/89 observations, 66.3%), while 15.7% (14/89 observations) of nets were stored and 2.2% (2/89 observations) being washed. The proportions lost, stored or being washed did not differ across the three surveys. Of the nets that were reported as being lost, 76.3% (45/59 observations) were destroyed, 22.0% (13/59 observations) stolen and 1.7% (1/59 observations) given to friends or family. It was common for children to share a bed net with siblings and 61.4% of children slept with three or more children. At the last survey in December, 62.2% (155/249) of ITNs were in good condition, 14.5% (36/249) were damaged and 23.3% (58/249) badly torn and non-protective. Net condition did not differ significantly by survey round, although there was a tendency towards net deterioration over the study period. Cone testing of a random sample of 26 children’s bed nets gave a mean knockdown at 1 h of 76.5% (95% CI = 61.9–91.1%) and mortality after 24 h of 42.2% (95% CI = 18.8–65.6%) for Olyset nets (9 nets, permethrin-treated) and mean knockdown of 85.2% (95% CI = 77.9–92.4%) and 24 h mortality of 39.9% (95% CI = 27.7–52.0%) for Permanet (17 nets, deltamethrin-treated).

**Fig. 3:**
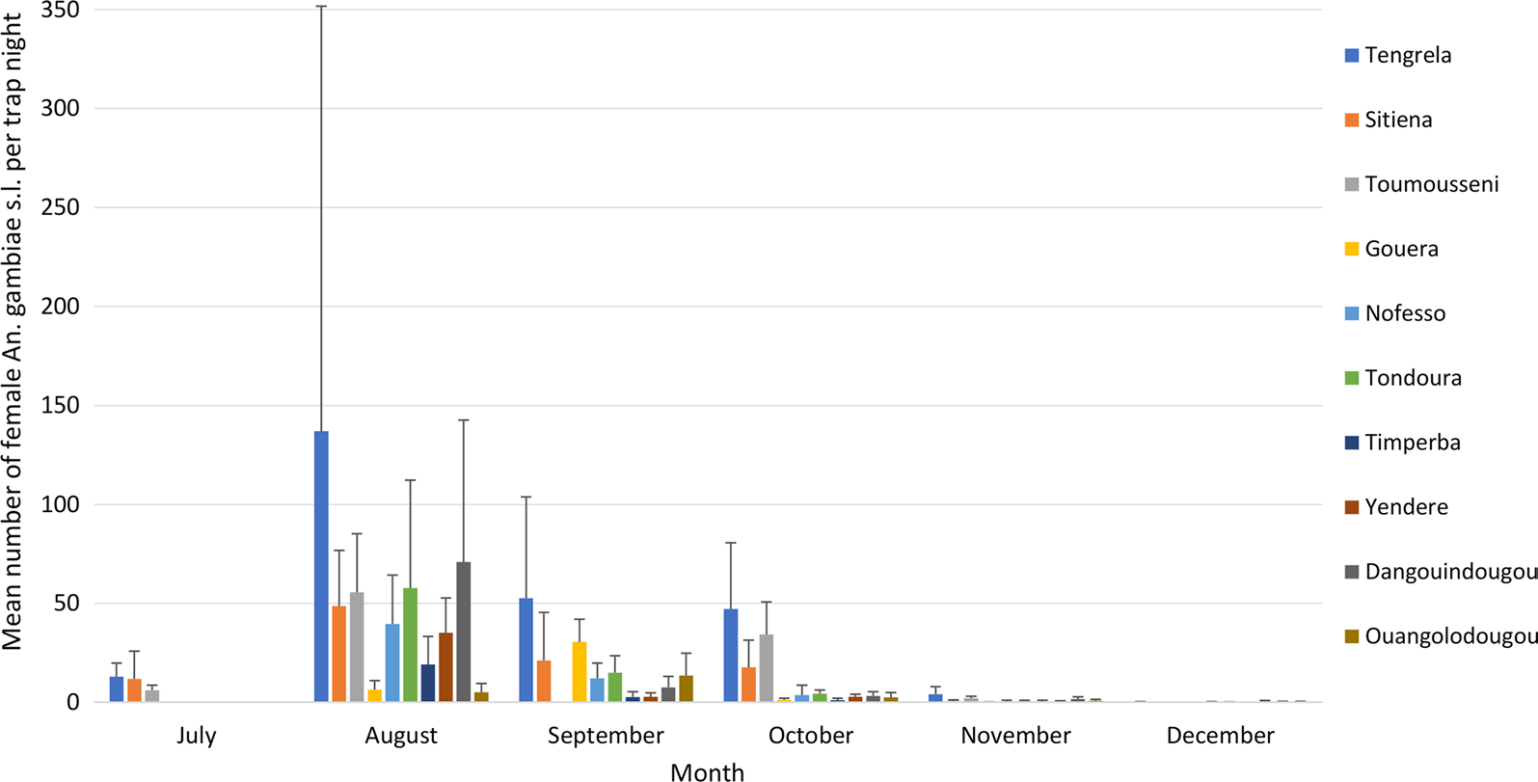
Mean number of *Anopheles gambiae s.l.* per trap night in sleeping rooms of study children during the transmission season

A total of 20,929 mosquitoes were caught from 1,151 trap collections with 16,270 of these being *An. gambiae s.l.*. Highest densities of *An. gambiae s.l.* occurred in August (Fig. 3). Tengrela village had the highest density of *An. gambiae s.l.* across the season, reaching a peak of 137 *An. gambiae s.l.* per trap /night in August. Of the 7,615 identified to species, 4,101 (53.9%) were *An. gambiae s.s*. and 2,590 (34.0%) *An. coluzzii.* 3.3% of the *An. gambiae s.l.* were sporozoite positive. The overall EIR in the study area was 80.4 infective bites/child over the six-month transmission season. The village-level EIR ranged from 40.8 in Timperba to 191.9 in Tondoura. Monthly systematic searches of study children’s bed nets in the early morning did not collect any mosquitoes. *Anopheles gambiae s.l.* were resistant to 0.05% deltamethrin in WHO tube tests with a mean corrected mortality of 52.3% (95% CI = 26.4–78.2%), with values ranging from 27.2% mortality in Toumousseni to 93.0% in Yendere.

The mean number of water bodies with anopheline larvae within 500 m of study houses was 8.7 (95% CI = 7.0–10.3). The average distance by road from the study house to the nearest clinic was 3.6 km (95% CI = 3.2–4.0), although for the villages of Toundoura and Gouera, which did not have a clinic nearby, the distance was 7.5 km and 9.4 km, respectively.

Univariate Poisson regression analysis identified two variables associated with incidence of *P. falciparum* infection in the study children and both remained significant in a multivariate model (Table 2). Although a small number of children travelled from the study area, this was associated with a 52% increase in the incidence of *P. falciparum* infection compared to children that did not travel (incidence rate ratio (IRR): 1.52, 95% CI: 1.45–1.59, *p* < 0.001). The least poor children were found to have a higher incidence of *P. falciparum* infection. A 1 unit increase in the SES factor score was associated with a 5% increase in infection rate (IRR: 1.05, 95% CI: 1.00–1.11, *p* = 0.04).

**Table 2:** Risk factors for *Plasmodium* infection incidence among children aged 5–15 years in Banfora region of Burkina Faso

Variables	Number of children	Number of infections	Time at risk (years)	Rate	Univariate analysis		Multivariate analysis	
					IRR (95% CI)	*P* value	IRR (95% CI)	*P* value
Age (years)								
5–7	76	164	31.09	5.28	–	–		
8–15	176	386	71.40	5.41	1.02 (0.94–1.12)	0.59		
Gender								
Male	131	288	52.93	5.44	–	–		
Female	121	262	49.56	5.29	0.97 (0.90–1.05)	0.46		
Ethnic group								
Other ethnicity	236	519	95.82	5.42	–	–		
Fulani	16	31	6.66	4.65	0.86 (0.65–1.13)	0.28		
Caregiver’s education								
Illiterate	199	443	80.45	5.51	–	–		
Literate	53	107	22.04	4.86	0.88 (0.76–1.02)	0.09		
Caregiver’s occupation								
Farmer	240	523	97.39	5.37	–	–		
Non farmer	12	27	5.09	5.30	0.99 (0.83–1.18)	0.89		
Religion								
Muslims	170	375	68.97	5.44	–	–		
Christians	33	67	13.93	4.81	0.88 (0.77–1.02)	0.09		
Animists	49	108	19.58	5.51	1.01 (0.87–1.18)	0.85		
SES quintile								
Poorest	46	102	20.16	5.06	1.04 (0.99–1.09)	0.14		
Poor	44	95	18.47	5.14				
Middle	44	98	18.21	5.38				
Rich	46	103	19.34	5.33				
Richest	47	106	17.79	5.96				
SES factor score								
1 unit increase	–	–	–	–	1.06 (1.01–1.11)	0.03	1.05 (1.00–1.11)*	0.04
Travel history during the study period								
No	246	537	100.93	5.32	–			
Yes	6	13	1.55	8.39	1.58 (1.31–1.90)	< 0.001	1.52 (1.45–1.59)^$^	< 0.001
Slept under bed net previous night								
Yes	203	448	83.55	5.36	–	–		
No	49	102	18.93	5.39	1.00 (0.78–1.29)	0.97		
Number of people sleeping in the child room (including child)								
≤ 6	55	124	23.10	5.37	–			
6 < no. ≤ 12	118	265	46.32	5.72	1.07 (0.94–1.21)	0.33		
> 12	79	161	33.06	4.87	0.91 (0.79–1.04)	0.16		
Bed net integrity (at final survey in December)								
Good (pHI: 0–64)	155	331	62.63	5.28	–			
Damaged (pHI: 65–642)	36	81	15.71	5.16	0.98 (0.83–1.15)	0.76		
Too torn (pHI: 643 +)	58	134	23.52	5.70	1.08 (0.97–1.20)	0.18		
Used other personal protective measures								
No	184	407	74.64	5.45	–	–		
Yes	58	125	24.53	5.10	0.93 (0.81–1.08)	0.36		
Housing: roof of child’s sleeping room								
Metal	191	420	77.60	5.41	–			
Non metal	52	114	21.85	5.22	0.96 (0.85–1.09)	0.57		
Housing: floor of the child’s sleeping room								
Cement/tiles	178	388	72.91	5.32	–	–		
Dirt	65	146	26.54	5.50	1.03 (0.94–1.13)	0.48		
Housing: wall of the child’s sleeping room								
Mud	65	151	26.54	5.69	–	–		
Brick	146	309	59.15	5.22	0.92 (0.83–1.02)	0.12		
Cement	32	74	13.77	5.37	0.94 (0.83–1.08)	0.39		
Housing: eaves of the child’s sleeping room								
Open	138	299	56.57	5.29	–			
Closed	102	229	41.60	5.51	1.04 (0.91–1.20)	0.57		
Cows, horses, sheep or goats within 5 m of child’s home								
Present	169	371	67.80	5.47	–			
Absent	80	174	33.51	5.19	0.95 (0.83–1.09)	0.46		
Euclidean distance to the nearest positive aquatic habitat								
< = 300 m	127	266	51.76	5.14	–	–		
> 300 m	125	284	50.72	5.60	1.09 (1.00–1.19)	0.06		
Distance by road to nearest health centre								
< = 2 km	119	252	49.65	5.08	–	–		
> 2 km	133	298	52.83	5.64	1.11 (1.00–1.24)	0.06		
EIR (village-level)								
-	–	–	–		1.00 (1.00–1.00)	0.08		
% mortality in WHO tube test with 0.05% deltamethrin diagnostic dose								
-	–	–	–	–	1.05 (0.73–1.50)	0.79		

*IRR* incidence rate ratio, *IRR adjusted for travel history, ^$^IRR adjusted for SES factor score

## Discussion

An extremely high incidence of *P. falciparum* infection was observed, with only three of the 252 cohort children remaining free from infection during the six-month follow-up period and 86.5% of children experiencing two or more infections. This high incidence of infection, despite regular retreatment with an effective anti-malarial if a child was infected, indicates the high force of infection in the study area and is supported by an estimated EIR of 80.4 infective bites per child during the transmission season. Similarly high levels of *P. falciparum* infection and malaria morbidity have been reported from other recent studies in Burkina Faso in areas of high ITN coverage [34–36].

The assessment of socio-demographic, entomological and environmental risk factors for *P. falciparum* infection did not elucidate any strong associations. This may be because of the overwhelming force of infection in the study area which meant that all the children were at extremely high risk of infection. Nevertheless, there was an indication of increased malaria risk in the few children that travelled overnight during the study period. Whether staying with family or for farming reasons, these children may be at higher risk of *P. falciparum* infection because they lack or have limited access to malaria prevention including ITNs, and diagnosis and treatment [37]. Overnight travel history was a risk factor for malaria in Uganda in a recent cohort study, with those not using ITNs at particularly high risk [38]. The finding of an association between higher SES factor score and a higher incidence of *P. falciparum* infection was unexpected since it is widely reported that the least poor children are typically at lower malaria risk than the most poor children [39]. The same pattern was also observed when SES quintiles were included in the model. The SES factor score was used in the multivariable model instead of the study quintiles since, unlike quintiles which allocate children into categories, the factor score better reflects the range of values in the dataset. The finding that the least poor children have a higher *P. falciparum* infection risk is not explained by a greater number of passively detected infections in these children. Passive cases contributed only 4% of all cases identified and there were no differences in this proportion across wealth quintiles. *Plasmodium falciparum* infection rates may be higher in the least poor children due to lower ITN use than the most poor children. Only 53.2% of the least poor children were reported to use an ITN the previous night compared to over 82.6% of children in the lowest quintile (most poor).

No association was found between *P. falciparum* infection risk and either of the two entomological variables hypothesized to impact on the primary outcome, namely EIR and insecticide resistance. The lack of association with EIR and vector density is counter-intuitive given it has been demonstrated in several other studies [40, 41], but could arise if vector densities in all villages were sufficiently high to maintain high transmission, or due to outdoor biting. Although levels of insecticide resistance varied between villages, the rate of *P. falciparum* infection was not greater in villages with higher levels of resistance. This finding may be partly due to the relatively small number of villages surveyed for insecticide resistance, and the resulting lack of statistical power, although no evidence of an association between malaria and insecticide resistance has been found in large area studies in the Sudan and Kenya [14]. Despite the high levels of pyrethroid resistance in the study area, no malaria vectors were found under the children’s ITN after 1,156 searches. This would imply that the nets are still providing some level of personal protection since a study in The Gambia found malaria mosquitoes under 48.3% of untreated bed nets following 1584 net searches [42]. Another study conducted in Kenya in an area of pyrethroid resistance found live *An. gambiae s.l.* in holed ITNs, with significantly higher numbers in nets with holes greater than 50 sq cm in size [43]. Although not measured in this cohort, early evening malaria vector biting is found in the study area (Sanou, pers. commun.). This is a time when it is common to find communities active in the peridomestic environment, for example cooking, eating or socializing. In the study site an estimated 85% of exposure to malaria vector bites can be prevented by use of ITNs from 22.00 to 05.00 but early evening biting outdoors and sporozoite rates of 3.3%, mean that residents are still exposed to ~ 32 infectious bites per person per year even with high ITN compliance (Sanou, pers. commun.). Early evening biting outdoors when people are active in the peridomestic environment is common across Africa [44], and highlights the need for vector control tools that can protect outdoors, such as insecticide-treated ‘eave ribbons’, attractive targeted sugar baits or larval source management [45–47].

ITN use was high with 80.6% of caregivers reporting that the study child slept under an ITN the previous night at the baseline survey. This is in line with other surveys including the 2017–8 MIS survey, which reported that 76.0% children under 5 years slept under an ITN the previous night [7]. The finding of lower reported ITN use in the least poor children (quintile 5) was unexpected. Other studies indicate higher ITN use among the least poor, including the 2018 Burkina Faso MIS survey in all ages which shows the least poor (quintile 5) have 1.4–1.6 times the odds of using an ITN the previous night compared to the most poor (quintile 1). It will be important to explore the reasons for the apparent lower ITN use among the least poor children in future studies. Data on ITN ownership or access was not collected as part of this study and so it is not known whether this was the cause of lower ITN use among the least poor children. Studies across SSA indicate that the net-use gap is primarily driven by intra-household access [48]. Despite the overall high reported bed net use in the study population, and considering that the NMCP campaign only took place the year before the study started, there was cause for concern about the durability of the ITNs since only 62.2% of the children’s ITNs were in good condition at the final survey. Although a longitudinal survey of the ITNs was not conducted, the finding is supported by studies from other countries. For example, in Tanzania a randomized double-blind prospective evaluation of the lifespan of three ITN products found that the functional survival (ITNs in serviceable condition) was 2 years for Olyset™ and 2.5 years for Permanet® ITNs [16], the two types of ITN delivered by the Burkina Faso NMCP. Bio-efficacy of the sample of children’s ITNs evaluated showed low 24-h mortality of 42.2% for Olyset™ nets and 39.9% for Permanet®, substantially below the ≥ 80% mortality threshold set by WHO. Thus, there is a great deal of uncertainty about net use and the protective efficacy of ITNs in Burkina Faso. The analysis shows that there was no difference in malaria risk between ITN users and non-users, nor that children sleeping under badly torn nets were more likely to have *P. falciparum* infections than those that slept under good nets.

The study has several limitations. Firstly, and most importantly, the study was probably underpowered to detect small risk factors. The sample size calculation assumed 50% prevalence of risk factors in the study population, while the study children were, in reality, relatively homogeneous with regard to risk factors shown to be important in other studies, for example, house construction. Secondly, while caregivers reported high compliance with ITN use, assessing ITN use is notoriously difficult and this may have impacted on the ability to identify this as an important risk factor. Indeed, social desirability bias and other forms of error mean that surveys are likely to substantially overestimate use [49], and objective and unobtrusive tools to measure ITN compliance are not currently available.

Burkina Faso is one of ten SSA countries designated as a High Burden High Impact country with a response plan including increased political will, strategic use of data to deploy tools for maximum impact and a multi-sectoral approach. While this study generates useful data on malaria burden in Banfora District, it is clear that additional tools will be needed to reduce this burden. Dual-active ITNs (pyrethroid plus piperonyl butoxide (PBO) or pyrethroid plus chlofenapyr) are now being deployed in the study area. PBO-pyrethroid ITNs have been shown to be more effective in reducing malaria than pyrethroid-only ITNs in areas of pyrethroid-resistant vectors [50, 51], and monitoring of the effectiveness of these dual active ITNs is ongoing in Burkina Faso. IRS should also be considered as it has been shown to be effective in reducing malaria in other high-burden countries. In an area of Uganda with an EIR of 176 infective bites/year, three rounds of IRS with the carbamate insecticide, bendiocarb, every six months reduced malaria incidence from 3.3 episodes to 0.6 episodes per person year [52]. Even this effective combination of interventions is, however, insufficient to eliminate malaria, so further interventions are required. SMC is currently being used in 12 sub-Sahelian countries, including Burkina Faso, in children up to 5 years of age, with a protective efficacy of over 50% against parasitaemia in Burkina Faso [22, 53]. A recent trial in Senegal found similarly high reductions in malaria when SMC was used in children aged under 5 years and in children aged 5 to 9 years [54]. Expanding the age range eligible for SMC could, therefore, be effective in reducing malaria further in Burkina Faso. Burkina Faso is also the site of pilot testing of gene drive mosquitoes, which if proven to be efficacious, feasible and acceptable, could be a potential option for malaria control in Burkina Faso.

## Conclusions

The study found overwhelmingly high levels of malaria transmission in Banfora district, in southwest Burkina Faso, and the risk factor survey did not identify any risk factors for further investigation to reduce the malaria burden. The findings have implications for achievement of the ambitious goals set out in the WHO Global Technical Strategy [55]. Malaria elimination in this area of intense seasonal transmission can only be achieved through the use of additional interventions.

## Data Availability

The datasets used and/or analysed during the current study are available from the corresponding author on reasonable request.

## Abbreviations

ACT: Artemisinin combination therapy
AL: Artemether-lumefantrine
CI: Confidence interval
CNRFP: Centre National de Recherche et de Formation sur le Paludisme
EIR: Entomological inoculation rate
IRR: Incidence rate ratio
IRS: Indoor residual spraying
ITN: Insecticide-treated net
NMCP: National Malaria Control Programme
PBO: Piperonyl butoxide
PCR: Polymerase chain reaction
pHI: Proportionate hole index
SD: Standard deviation
SES: Socio-economic status
SMC: Seasonal malaria chemoprevention
SSA: Sub-Saharan Africa
WHO: World Health Organization
